# Streamlining Patient Transitions: A Surgical Discharge Card Initiative at Almanagil Teaching Hospital

**DOI:** 10.7759/cureus.90125

**Published:** 2025-08-14

**Authors:** Abubakr Muhammed, Abdalmahmoud Asadig Kanan Ahmed, Mohammad Alrawi, Mohamed Elhassan Momin Mohamed Elhassan Noreldayem, Mohamed Shamseldin Elsiddig Mohamed, Asma Ahmed Osman Mohamed, Marwa Yousif, Nahla Widatalla Abdalla Hamrawi, Suliman Saadeldeen, Maria Faisal Abdalwahab Ali, Rania Yasser Babiker Mansour, Ijlal Eltaiyb Ali Mohammed, Mohamed Abdalrhman Nour Ahmed, Mohamed Kamalaldein Hamad Mohamednour, Manhal Eisa Galal Eisa, Mohey Aldien Ahmed Elamin Elnour, Afan Hussein, Hana Barid Elnisma, Yousra Goriesh AlNoor Sidahmed, Altayeb Yousif Abdelgadir Mohammed

**Affiliations:** 1 Surgery, University of Gezira, Madani, SDN; 2 General Surgery, Managil Teaching Hospital, Managil, SDN; 3 Surgery, Burjeel Medical City, Abu Dhabi, SAU; 4 General Surgery, Sudan Medical Specialization Board, Khartoum, SDN; 5 Neurosurgery, University Hospitals Coventry and Warwickshire NHS Trust, Coventry, GBR; 6 General Practice, Jorvik Gillygate Practice, York, GBR

**Keywords:** discharge card standardization, discharge card standardization, documentation compliance, patient transitions, postoperative continuity

## Abstract

Background: Inadequate discharge documentation at Almanagil Teaching Hospital posed significant risks to patient safety and continuity of care, consistent with challenges seen in similar healthcare settings. The hospital aimed to address these gaps by enhancing the completeness, accuracy, and clarity of surgical discharge documentation through the development and implementation of a standardized discharge card, coupled with targeted staff training.

Methods: A prospective quality improvement project was conducted in two cycles (May-June 2025), involving audits of 44 (First Cycle) and 51 (Second Cycle) surgical discharge cards. Following baseline assessments, a structured discharge card was developed and implemented, along with targeted clinical staff training.

Results: Post-intervention audits revealed substantial improvements. Documentation of telephone number and address rose from 0 (0%) to 47 (92.2%) and 49 (96.1%), respectively. The number of hospital file entries increased from 29 (65.9%) to 49 (96.1%). Referrers' names, roles, organizations, and contact details improved from less than three (6.8%) to 51 (100%). Clinical elements, such as documentation of intraoperative and postoperative complications, rose from nine (20.5%) and eight (18.2%) to 51 (100%). Overall compliance increased from 52.9% to 94.6%, marking a 41.7% gain.

Conclusion: The intervention significantly enhanced discharge documentation quality, reinforcing standardization, patient safety, and accountability. The model is scalable to similar resource-limited settings and warrants sustained auditing and ongoing training for long-term impact.

## Introduction

Hospital discharge documentation is a vital component of patient care, ensuring continuity and safety as patients transition from hospital to community-based settings [1]. High-quality discharge summaries facilitate effective communication between hospital staff and primary care providers, reducing the risk of medication errors, readmissions, and adverse health outcomes [1,2]. In resource-limited environments such as Sudan, the quality of discharge cards is often compromised by incomplete or unclear documentation, which can lead to significant gaps in patient care [3-6].

Recent clinical audits at Sudanese hospitals have revealed persistent deficiencies in discharge documentation, including missing or inaccurate information about admission and discharge dates, clinician names, and medication changes [6]. For example, at Al-Shaab Hospital, the majority of discharge forms lacked essential details such as the date of admission and the name of the responsible doctor [6]. Similar challenges have been identified in other Sudanese hospitals, where audits have shown that only a minority of discharge cards meet basic standards for completeness and legibility [5,6].

These issues are not unique to Sudan. International studies have demonstrated that incomplete or inaccurate discharge summaries are a widespread concern, even in countries with advanced healthcare systems [6,7]. In the United Kingdom, audits have shown that adherence to national guidelines for discharge information is often suboptimal, particularly regarding the documentation of medication changes and the reasons for those changes [7]. Medication errors pose a particular risk, with studies indicating that both manual and electronic discharge summaries are susceptible to transcription errors and omissions [7-9].

Quality improvement initiatives have demonstrated that standardized discharge card templates, regular staff training, and ongoing clinical audits can significantly enhance the quality of discharge documentation [10-13]. In Sudan, the introduction of standardized discharge cards and targeted education for healthcare providers has led to marked improvements in documentation completeness and accuracy. International experience also supports the value of such interventions, with studies showing that structured templates and regular feedback can improve the timeliness and content of discharge summaries [12-15].

Given these findings, there is a clear need to improve the quality of discharge cards at Almanagil Teaching Hospital. Standardizing the discharge card format, providing training for healthcare staff, and implementing regular audits are essential steps to ensure that patients receive clear, comprehensive, and actionable discharge instructions. These measures will help to reduce the risk of adverse events, support safer transitions from hospital to community-based care, and ultimately improve patient outcomes.

## Materials and methods

Study design and setting

This prospective quality improvement project (QIP) was conducted at Almanagil Teaching Hospital, a tertiary care center in Sudan serving a diverse urban and rural population. The project targeted the surgical department, where discharge documentation was identified as an area for improvement. The QIP began on May 10, 2025, and is ongoing, with the initial intervention and evaluation phases completed by June 30, 2025.

Study phases and interventions

First Cycle (Pre-intervention State and Root Cause Analysis: May 25, 2025-June 6, 2025; Duration: 13 Days)

Baseline data collection: Baseline data were collected by reviewing existing discharge documentation practices in the surgical department. A structured checklist, based on national and international standards [1], was used to assess the presence and completeness of key discharge information, including patient identifiers, clinical summaries, medication lists, and follow-up instructions.

Problem identification: The baseline assessment revealed that discharge records lacked standardization and frequently omitted essential details, including follow-up instructions, medication lists, and patient identifiers. Some clinicians did not clearly document clinical summaries or post-discharge plans.

Root cause analysis: Through direct observation and stakeholder interviews (physicians, nurses, administrators, and patients), the team identified the absence of a standardized discharge card, inconsistent documentation practices, and limited staff awareness as major contributors to these deficiencies.

Intervention (Development and Implementation: June 7, 2025-June 20, 2025; Duration: 14 Days)

Development of a standardized discharge card: A new standardized discharge card was developed collaboratively with medical staff and hospital management. The Surgical Discharge Card, introduced at Almanagil Teaching Hospital, is a rigorously structured documentation instrument designed to facilitate standardized, accurate, and comprehensive communication during patient transitions following surgical procedures. The card is methodically organized into two distinct panels, the front page and the back page, each comprising dedicated subheadings that reflect the sequential stages of clinical care.

**Figure 1:**
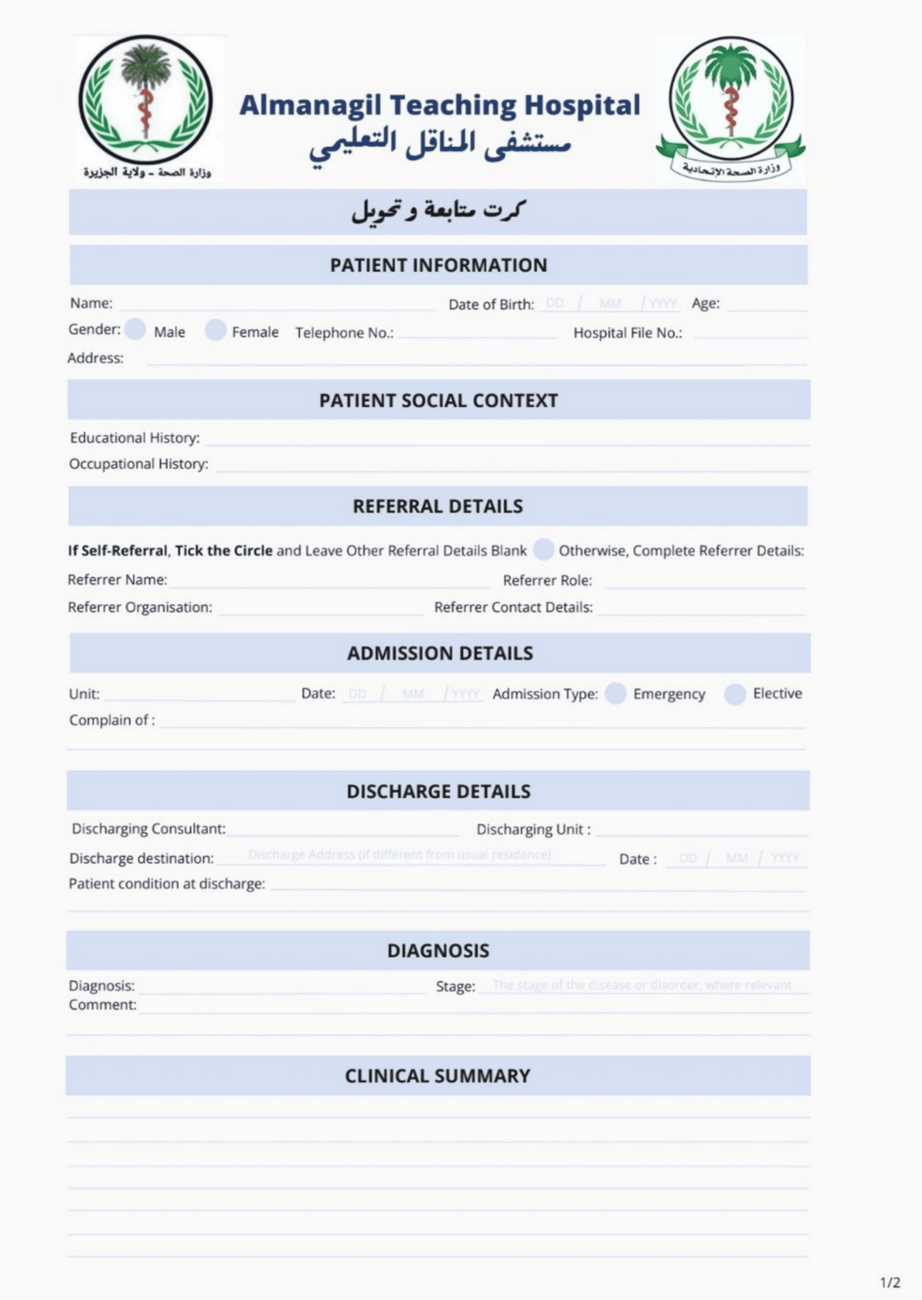
Front Page of the New Surgical Discharge Card

The front page (Figure 1) includes three key subheadings: Patient Identification, Referral and Admission Information, and Personal and Social Context. These sections collectively capture essential personal demographics, the source and nature of the patient's admission, and relevant social or contextual data necessary for understanding the patient's background.

**Figure 2:**
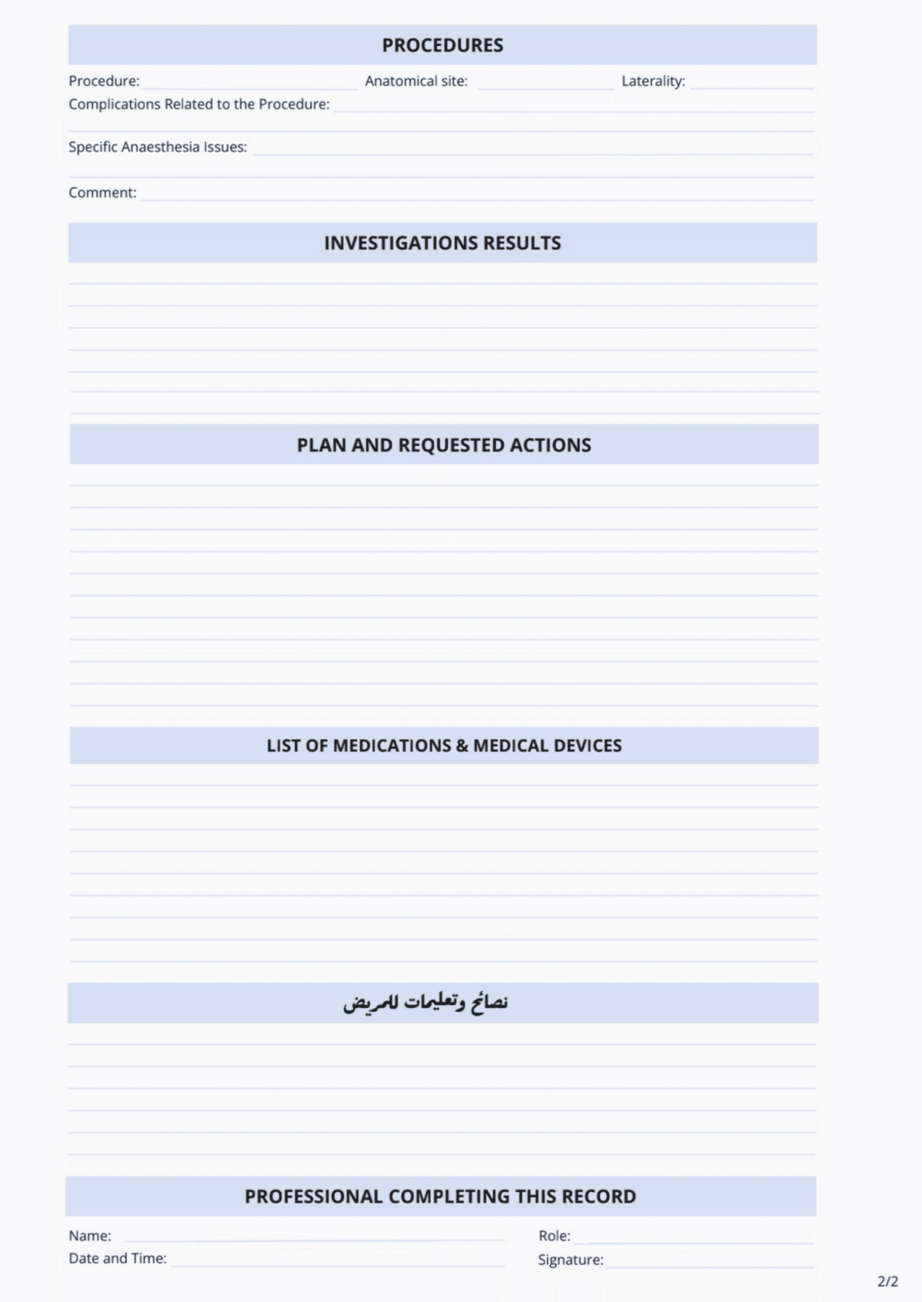
Back Page of the New Surgical Discharge Card

The back page (Figure 2) is composed of four subheadings: Clinical Summary, Postoperative Recommendations and Instructions, Patient Communication, and Record Authentication. These components document the surgical findings, discharge medications, planned follow-up care, postoperative advice, and formal validation by responsible professionals.

Training and support: All healthcare professionals involved in discharge documentation (doctors and nurses) received structured training sessions. These included didactic instruction, hands-on practice with sample scenarios, and reinforcement of the importance of accurate and complete documentation. Visual aids, posters, and quick-reference guides were distributed in clinical areas to promote ongoing awareness and compliance. Follow-up training was provided during the first month.

Implementation: The standardized discharge card was introduced in the surgical department, and compliance was monitored in real time.

Second Cycle (Post-intervention State and Evaluation: June 21, 2025-June 30, 2025; Duration: 10 Days)

Evaluation of the new system: After implementation, discharge cards were reviewed for completeness and accuracy using the same structured checklist. Compliance with the new format was measured, and feedback was collected from both healthcare providers and patients to identify strengths, weaknesses, and areas for further improvement. Additional training was provided as needed, based on feedback and observed documentation gaps. Necessary adjustments were made to the discharge card and the implementation process to enhance usability and alignment with clinical needs.

Data analysis

Sampling

Discharge cards were randomly selected from both pre- and post-intervention periods (First Cycle N = 44, Second Cycle N = 51).

Assessment Tools and Criteria

Three trained physicians independently evaluated the discharge cards for the presence of key data elements, using a standardized protocol to ensure consistency. The proportion of "yes" responses for each checklist item was calculated for both cycles, and percentage improvement was determined.

Statistical Analysis

Descriptive statistics were used to summarize the completeness of documentation. Qualitative feedback from staff and patients was analyzed to guide further refinements. While randomization and trained reviewers strengthened reliability, the absence of blinding may introduce bias.

Evaluation

Feedback and Effectiveness Assessment

The impact of the intervention was assessed by comparing documentation completeness and compliance rates before and after the introduction of the standardized discharge card. Structured feedback from healthcare providers and patients was used to identify ongoing challenges and inform future improvements.

Ethical considerations

Ethical and managerial approval for the QIP was obtained from Almanagil Teaching Hospital. All personal and clinical data were anonymized to protect patient privacy and confidentiality.

## Results

A total of 95 discharge cards were reviewed during the study: 44 from the First Cycle and 51 from the Second Cycle. The implementation of the standardized discharge card led to significant improvements in the completeness, accuracy, and clarity of discharge documentation across nearly all assessed domains (Table 1).

**Table 1:** Cycle-Based Compliance Comparison for Surgical Discharge Documentation

Question	First Cycle (N = 44)	Second Cycle (N = 51)	Percentage Improvement
Is the patient’s name recorded	44 (100.0%)	51 (100.0%)	0.0%
Is the age specified	43 (97.7%)	51 (100.0%)	2.3%
Is the gender indicated	23 (52.3%)	51 (100.0%)	47.7%
Is the telephone number listed	0 (0.0%)	47 (92.2%)	92.2%
Is the hospital file number noted	29 (65.9%)	49 (96.1%)	30.2%
Is the address included	0 (0.0%)	49 (96.1%)	96.1%
Is self-referral status indicated	2 (4.5%)	21 (41.2%)	36.6%
Is the referrer’s name entered	2 (4.5%)	51 (100.0%)	95.5%
Is the referrer’s role described	3 (6.8%)	51 (100.0%)	93.2%
Is the referrer’s organization named	1 (2.3%)	51 (100.0%)	97.7%
Are contact details for the referrer provided	2 (4.5%)	51 (100.0%)	95.5%
Is the admitting unit identified	34 (77.3%)	51 (100.0%)	22.7%
Is the admission date filled in	43 (97.7%)	48 (94.1%)	-3.6%
Is the type of admission (Emergency/Elective) mentioned	1 (2.3%)	51 (100.0%)	97.7%
Was the patient’s complaint listed	35 (79.5%)	50 (98.0%)	18.5%
Is the name of the discharging consultant included	17 (38.6%)	49 (96.1%)	57.4%
Is the discharging unit stated	27 (61.4%)	49 (96.1%)	34.7%
Is the discharge destination mentioned	2 (4.5%)	35 (68.6%)	64.1%
Is the discharge address (if different) noted	0 (0.0%)	18 (35.3%)	35.3%
Is the discharge date captured	40 (90.9%)	47 (92.2%)	1.2%
Is the patient’s condition at discharge clarified	27 (61.4%)	50 (98.0%)	36.7%
Is a diagnosis entered	43 (97.7%)	51 (100.0%)	2.3%
Is the stage of the condition specified	3 (6.8%)	49 (96.1%)	89.3%
Are any diagnostic comments included	9 (20.5%)	50 (98.0%)	77.6%
Are the patient’s complaints outlined	35 (79.5%)	51 (100.0%)	20.5%
Is the procedure clearly listed	36 (81.8%)	51 (100.0%)	18.2%
Is the date of the procedure mentioned	39 (88.6%)	50 (98.0%)	9.4%
Is the anatomical site referenced	16 (36.4%)	50 (98.0%)	61.7%
Is laterality (left/right) indicated	12 (27.3%)	38 (74.5%)	47.2%
Are intraoperative complications reported	9 (20.5%)	51 (100.0%)	79.5%
Are postoperative complications noted	8 (18.2%)	51 (100.0%)	81.8%
Are the findings of investigations available	2 (4.5%)	46 (90.2%)	85.7%
Is a post-care plan or action requested	25 (56.8%)	51 (100.0%)	43.2%
Are patient instructions and advice clearly provided	10 (22.7%)	51 (100.0%)	77.3%
Is the location of the next follow-up visit stated	22 (50.0%)	50 (98.0%)	48.0%
Is the date of the next follow-up appointment specified	24 (54.5%)	51 (100.0%)	45.5%
Is the professional’s name provided	11 (25.0%)	50 (98.0%)	73.0%
Is their role outlined	1 (2.3%)	45 (88.2%)	86.0%
Is the date and time of entry included	14 (31.8%)	41 (80.4%)	48.6%
Was the signature included	13 (29.5%)	46 (90.2%)	60.7%

The QIP at Almanagil Teaching Hospital demonstrated a remarkable enhancement in surgical discharge documentation practices. Overall compliance rose from 52.9% in the initial phase to 94.6% in the follow-up phase, reflecting a 41.7% improvement. This substantial progress was driven by structured interventions, including the development of a standardized Surgical Discharge Card and targeted education for clinical staff. Components that previously showed minimal documentation, such as contact details, referrer information, and postoperative data, reached near-perfect completion rates. The initiative underscores the hospital's commitment to patient safety, data accuracy, and alignment with international standards through practical, sustainable improvement strategies (Table 2).

**Table 2:** Overall Compliance Improvement in Surgical Discharge Card Quality

Audit Cycle	Overall Compliance Rate
First Cycle	52.9%
Second Cycle	94.6%
Improvement	+41.7%

Improvements in documentation completeness

Personal and Administrative Data

Prior to the intervention, documentation of basic patient identifiers was inconsistent. While the patient's name was universally recorded in both cycles, 44 (100%) and 51 (100%), other key identifiers such as age, gender, address, and hospital file number were frequently omitted in the First Cycle. For example, the inclusion of the patient's telephone number and address improved dramatically from 0 (0%) pre-intervention to 47 (92.2%) and 49 (96.1%) post-intervention, respectively. The recording of the hospital file number increased from 29 (65.9%) to 49 (96.1%).

Referrer and Admission Details

The documentation of the referrer's name, role, organization, and contact details showed the most striking improvement, rising from less than three (6.8%) in the First Cycle to 51 (100%) in the Second Cycle for most items. Similarly, the identification of the admitting unit and type of admission (emergency/elective) improved from 34 (77.3%) and one (2.3%) to 51 (100%) for both items post-intervention.

Clinical Data

The completeness of clinical information also improved substantially. Documentation of gender increased from 23 (52.3%) to 51 (100%), and the recording of the patient's complaint, diagnosis, and procedure details all reached or approached 51 (100%) completeness after the intervention. Notably, the inclusion of intraoperative and postoperative complications increased from nine (20.5%) and eight (18.2%) to 51 (100%), respectively. The percentage of records with the stage of the condition specified rose from three (6.8%) to 49 (96.1%).

Discharge and Follow-up Planning

Post-discharge planning documentation saw marked improvement. The inclusion of follow-up instructions, patient advice, and the date/location of the next appointment all improved significantly, with most fields reaching 51 (100%) completeness in the Second Cycle. The documentation of the discharge destination and address (if different) also increased, though these remained less than 70% in the Second Cycle, indicating ongoing areas for improvement.

Professional and Authentication Details

The documentation of the professional's name, role, date/time of entry, and signature improved substantially, with most items exceeding 80% completeness post-intervention. For example, the inclusion of the professional's role increased from one (2.3%) to 45 (88.2%), and the presence of a signature rose from 13 (29.5%) to 46 (90.2%).

## Discussion

The implementation of a standardized discharge card and structured training at Almanagil Teaching Hospital led to substantial improvements in the completeness and accuracy of discharge documentation. This aligns with both national and international evidence that supports the value of structured templates, staff education, and regular audits in enhancing discharge summary quality, especially in resource-limited settings [1-3].

Prior to the intervention, discharge documentation was frequently incomplete, with essential elements such as patient identifiers, clinical summaries, and follow-up instructions often omitted. This is consistent with findings from other Sudanese hospitals, where audits have revealed persistent deficiencies in discharge documentation, including missing or inaccurate information about admission and discharge dates, clinician names, and medication changes [5,6]. For example, Eissa et al. found that most discharge summaries at Al-Shaab Hospital lacked key details such as the date of admission and the name of the responsible doctor [6]. Similar challenges have been identified in other Sudanese hospitals, where only a minority of discharge cards met basic standards for completeness and legibility [6].

These issues are not unique to Sudan. International studies have demonstrated that incomplete or inaccurate discharge summaries are a widespread concern, even in countries with advanced healthcare systems [7]. In the United Kingdom, audits have shown that adherence to national guidelines for discharge information is often suboptimal, particularly regarding the documentation of medication changes and reasons for those changes [7]. Medication errors are a particular risk, with studies showing that both manual and electronic discharge summaries are prone to transcription errors and omissions [7,8].

Following the introduction of the standardized discharge card and associated training, the completeness of documentation improved dramatically. For example, documentation of the patient's telephone number and address improved from 0 (0%) pre-intervention to 47 (92.2%) and 49 (96.1%) post-intervention, and the recording of the hospital file number increased from 29 (65.9%) to 49 (96.1%). The documentation of the referrer's name, role, organization, and contact details rose from less than three (6.8%) to 51 (100%) post-intervention. Clinical data, including intraoperative and postoperative complications, also improved from nine (20.5%) and eight (18.2%) to 51 (100%), respectively. These results are consistent with other quality improvement efforts, where the use of structured templates and regular audits has led to significant gains in both the content and clarity of discharge summaries [8].

The improvement in documenting medication information and follow-up plans is particularly noteworthy, as these elements are critical for patient safety and continuity of care [8]. Studies have shown that incomplete discharge summaries contribute to medication errors and adverse events after hospital discharge [8-10]. By ensuring that discharge cards consistently include medication changes, follow-up appointments, and clear instructions, this project directly addresses a key patient safety concern.

Active staff engagement, ongoing training, and real-time feedback were critical to the success of this intervention. Visual aids and quick-reference guides supported compliance, while periodic refresher sessions reinforced best practices. These strategies align with recommendations from the literature, which emphasize the importance of multidisciplinary collaboration and continuous education in sustaining quality improvements [12-14].

Despite substantial progress, some areas, such as documentation of discharge destination and address, remained below optimal levels in the post-intervention phase, indicating the need for further refinements to the discharge card and additional training. The absence of blinding in the evaluation process may have introduced observer bias, and the relatively short follow-up period limits the assessment of long-term sustainability. The lack of explicit documentation of informed consent is another limitation, although patient confidentiality was maintained. Future projects should address this to strengthen ethical compliance.

This project provides a scalable model for improving discharge documentation in other departments and hospitals across Sudan. Standardizing discharge processes, investing in staff training, and implementing regular audits are practical steps that can be widely adopted to enhance patient safety and reduce preventable readmissions [12-14]. Policymakers and hospital administrators should prioritize these interventions to improve patient outcomes.

Limitations

This study was conducted within a single surgical department at a tertiary care center, which may limit generalizability to other specialties or institutions. The short evaluation period prevented assessment of long-term sustainability and staff adherence over time.

## Conclusions

In conclusion, the structured approach adopted at Almanagil Teaching Hospital significantly improved the quality and completeness of discharge documentation, aligning with both national and international best practices. Sustained attention to training, auditing, and stakeholder engagement will be essential to maintain and build on these gains, ultimately supporting safer transitions of care and better patient outcomes.
